# PepPSy: a web server to prioritize gene products in experimental and biocuration workflows

**DOI:** 10.1093/database/baw070

**Published:** 2016-05-12

**Authors:** Olivier Sallou, Paula D. Duek, Thomas A. Darde, Olivier Collin, Lydie Lane, Frédéric Chalmel

**Affiliations:** ^1^Genouest Bioinformatics Platform, IRISA, Campus de Beaulieu, Rennes 35042, France; ^2^CALIPHO Group, SIB Swiss Institute of Bioinformatics, CMU, Michel Servet 1, Geneva 1211, Switzerland; ^3^IRSET, Inserm U1085, 9 avenue du Professeur Léon Bernard, Rennes 35000, France; ^4^Department of Human Protein Sciences, Faculty of Medicine, University of Geneva, CMU, Michel Servet 1, Geneva 1211, Switzerland

## Abstract

Among the 20 000 human gene products predicted from genome annotation, about 3000 still lack validation at protein level. We developed PepPSy, a user-friendly gene expression-based prioritization system, to help investigators to determine in which human tissues they should look for an unseen protein. PepPSy can also be used by biocurators to revisit the annotation of specific categories of proteins based on the ‘omics’ data housed by the system. In this study, it was used to prioritize 21 dubious protein-coding genes among the 616 annotated in neXtProt for reannotation. PepPSy is freely available at http://peppsy.genouest.org.

**Database URL:** http://peppsy.genouest.org.

## Introduction

Analysis of the human genome led to the identification of approximately 20 000 protein-coding genes, which produce a variety of functional proteoforms via different mechanisms including genetic polymorphisms, alternative splicing, post-translational modifications, or processing. The Human Proteome Project (HPP) launched by the Human Proteome Organization (HUPO) aims at providing experimental validation for these proteoforms and understanding their role in health and disease (1). neXtProt is an innovative knowledge platform focusing on human proteins, that is built on top of UniProtKB/Swiss-Prot annotations (2) and provides additional expert-curated information on protein expression, subcellular localization, post-translational modifications and protein variations, gathered from selected high-throughput datasets (3). Whenever possible, neXtProt provides links to primary data providers, such as the Human Protein Atlas (HPA) (4). It is also cross-referenced to the main expert-curated sequence knowledgebases UniProt (2) and RefSeq (5), and to integrative databases such as GeneCards (6) that automatically collect available information on human genes. Since neXtProt has been chosen as the reference database for the HPP, it collects and displays all the mass spectrometry and antibody-based data generated by the HPP consortium (7).

Since 2008, UniProtKB has been assigning a ‘protein existence’ (PE) score to each entry, ranging from PE1 (entry with evidence at protein level) to PE5 (uncertain). PE5 entries correspond to dubious protein sequences that are kept in UniProtKB/Swiss-Prot until other major genome annotation resources involved in the CCDS project (8) reach a consensus and annotate them either as coding or as noncoding. If the sequence is annotated as noncoding, the corresponding entry is removed from UniProtKB and stored in UniParc (9). If it is annotated as coding, then it can take a PE score of 1 (existence validated at protein level), 2 (existence validated at transcript level), 3 (existence validated by homology), or 4 (existence validated by a gene model), depending on the available information. neXtProt uses the same classification and upgrades PE2, PE3 and PE4 entries to PE1 when there is clear mass spectrometry evidence for the existence of the corresponding gene products. According to the standard metrics table of the neXtProt database (release 2014-09-19), there are still about 3000 genes that were classified as coding but for which none of the products could be validated at protein level (3). Most of these proteins escaped detection either because their physico-chemical properties are not compatible with mass-spectrometry or antibody-based techniques, or because their expression pattern is restricted in time or space. One of the first objectives of the Chromosome-centric Human Proteome Project (C-HPP) is to validate the existence of those so-called ‘missing’ proteins corresponding to neXtProt entries annotated with PE scores ranging from PE2 to PE4 (7).

Customizable workflows, such as CAPER 2.0 (10), have already been used to discover novel genes by integrating transcriptomic and proteomic data (11). In the HPP context, the use of transcriptomics datasets has also proven to be valuable to determine where to look for missing proteins (12–14). To the best of our knowledge, there is no dedicated and freely-accessible workflow to assist researchers in prioritizing missing proteins for detection, and suggesting potential biological samples. We thus developed PepPSy, a user-friendly gene expression-based system aiming at identifying the tissues in which unseen gene products should be looked for. PepPSy currently embeds eight filtration criteria and seven prioritization modules dedicated to the filtration of protein candidates and their ranking according to gene expression-based information.

Since most PE5 entries correspond to erroneous translations of non-protein-coding elements, they are not expected to be detected in proteomics experiments. Therefore, any claim for a PE5 protein identification is a priori considered as artefactual and needs to be carefully documented (15). However, recent publications highlighted that a few of them might still be true proteins (16,17). In order to get a chance to be validated by proteomics, it is crucial that these entries are kept in UniProtKB, the safest way being by upgrading their PE status to PE2-4. We have applied PepPSy to revisit the annotation of PE5 entries based on existing expression information. As a result, 21 entries have been provided transcriptomic evidence for further validation and reclassification by neXtProt and UniProt, and eight entries have been reclassified.

## PepPSy data and interface

### The ‘*Filtration*’ tab

**Figure 1.**
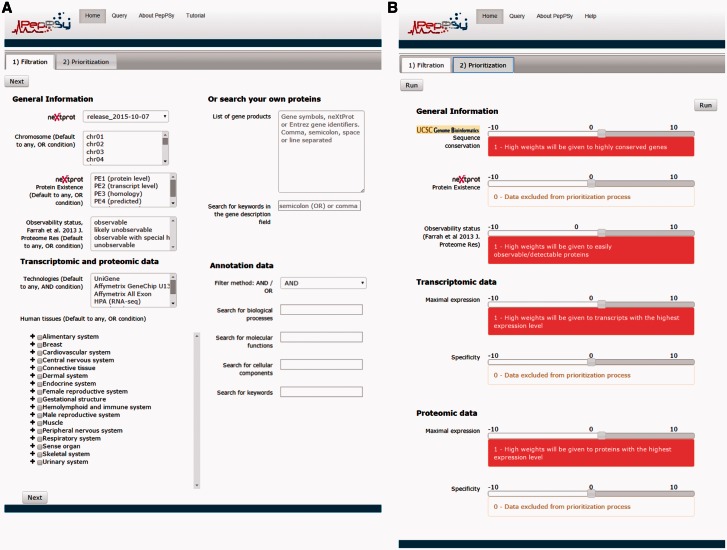
PepPSy layouts. (**A**) The ‘Filtration’ and (**B**) the ‘Prioritization’ tab contain menus to filter proteins and sliders to increase (red, from +1 to +10) or decrease (blue, from -10 to -1) contribution of each prioritization module to the final ranking.

PepPSy has a simple and intuitive interface including a ‘*Filtration*’ tab (Figure 1A) which enables users to apply up to eight selection criteria from menus organized into four categories: *General information*, *Transcriptomic and proteomic data*, *Annotation data* and *Search your own proteins*. In the former category, the user can select candidates according to: (i) the neXtProt database release; (ii) the chromosome location provided by neXtProt; (iii) the neXtProt protein existence (PE) status; and, (iv) the observability status (ranging from ‘observable’ to ‘unobservable’) according to the Farrah *et al.* classification that is supposed to reflect the probability to identify a given protein based on several parameters (18). In the *Transcriptomic and proteomic data* category, PepPSy allows users to filter candidate gene products based on (v) their presence in six distinct transcriptomic and proteomic datasets including: the NCBI UniGene/EST dataset (45 human experimental conditions (HECs), such as tissues, organs or cell types) (5); an Affymetrix 3′ Gene array dataset (109 HECs; Supplementary Table S1) (19); an Affymetrix All Exon array dataset containing 12 human tissues (raw data available on manufacturer's website at http://www.affymetrix.com/support/technical/sample_data/exon_array_data.affx); the Illumina RNA-sequencing dataset (32 HECs) of the Human Protein Atlas (HPA) database (4); the antibody-based protein expression profiling (83 HECs) of HPA (4); and a LC/MS-based protein expression profiling (30 HECs) (20). One can also directly select candidates expressed in (vi) specific anatomical systems or tissues. In the corresponding menu, HECs that are analysed in at least one of the 6 transcriptomic and proteomic datasets are classified using the human anatomy ontology provided by the neXtProt platform (ftp://ftp.nextprot.org/pub/current_release/controlled_vocabularies/caloha.obo). Briefly, the menu contains 2 levels in which 60 human tissues/organs (level 2) are classified in 17 major systems of the human body (level 1).

The *Annotation data* category allows users to select candidates according to (vii) their associated annotations including biological process, molecular function and cellular component GO terms as well as keywords that are searched in the ‘Keyword’ field of each entry (21) as provided by neXtProt (3). Finally, (viii) a custom list of gene products can be queried using the text field of the latter category termed ‘*Search your own proteins’*. The User is also able to select candidates by searching keywords in the ‘description’ field of each entry. Currently, PepPSy only accepts neXtProt and NCBI’s Entrez Gene identifiers as well as gene symbols. For other database entry conversions, users are referred to the UniProt's online conversion service available at http://www.uniprot.org/uploadlists (9).

### The ‘*Prioritization*’ tab

PepPSy provides a direct access to seven different ranking modules in a ‘Prioritization’ tab organized into three categories (Figure 1B), i.e. *General Information*, *Transcriptomic data* and *Proteomic data*.

The *General Information* category contains three modules which allow to obtain a ranked list of the neXtProt entries according to: (i) their degree of evolutionary sequence conservation across vertebrate genomes by averaging the base-by-base phastCons conservation scores calculated among 100 vertebrate species as provided by the UCSC genome browser (22); (ii) their neXtProt PE status; and, (iii) their observability status as defined by Farrah and collaborators (18).

Both *Transcriptomic data* and *Proteomic data* categories include two modules, *Maximal expression* and *Specificity*, which rank neXtProt gene products according to their maximal abundance at the (iv) transcript- and (v) protein-levels by using the transcriptomic and proteomic datasets described in the ‘Filtration’ tab; and to their restricted expression pattern across human tissues at the (vi) transcript- and (vii) protein-levels as defined by the Shannon entropy Q (categorical) (19,23).

Importantly, the interface of the *Prioritization* tab enables users to apply higher or lower weights to each prioritization module thus increasing or decreasing its contribution to the final ranking algorithm. By taking the weight applied to the *Specificity* module of the *Transcriptomics data* category as an example: a high positive weight (from +1 to +10) will tend to give a better ranking to gene products displaying an expression at the transcript-level (based on the UniGene/EST, the Affymetrix 3′ Gene array, the Affymetrix All Exon array and/or the Illumina RNA-sequencing transcriptomic datasets) restricted to only few human tissues than to gene products ubiquitously detected in all organs; a low negative weight (from -10 to -1) will give a high final rank to ubiquitously-expressed genes; finally, a weight of 0 means that this module will be not taken into account to compute the overall ranking. Another example: a high positive weight (from +1 to +10) to the *Sequence conservation* module will tend to give high importance in the overall ranking to neXtProt entries associated with protein sequence conserved across 100 vertebrate species.

**Figure 2.**
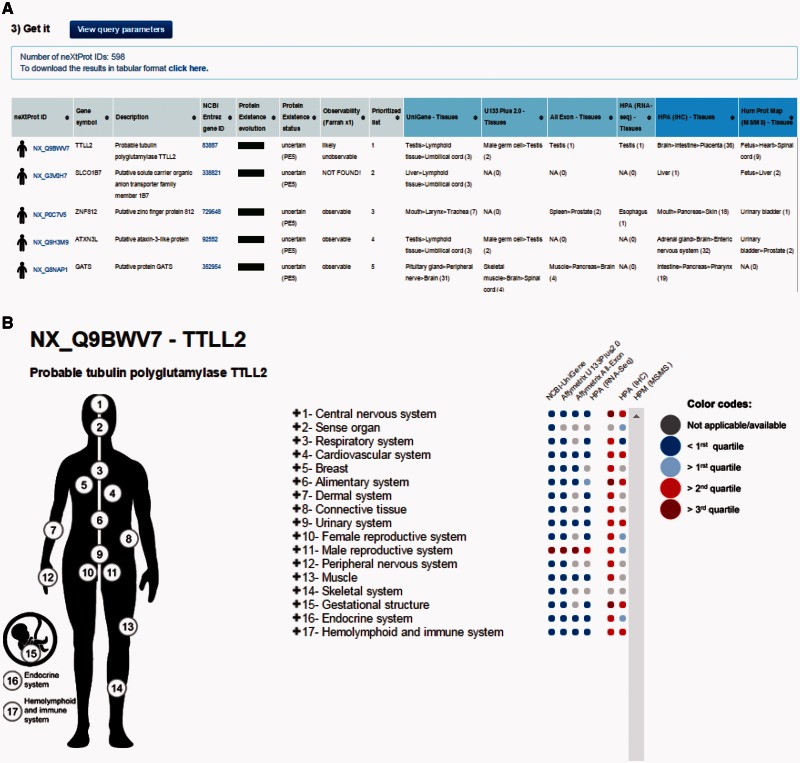
PepPSy outputs. (**A**) The output displays columns for neXtProt IDs, gene symbols, protein description, NCBI Entrez gene IDs, a color-coded evolution of the neXtProt Protein Existence status over time, the current Protein Existence status, the *observability* status, the prioritized rank of each gene product and the human tissues in which ranked genes are expressed in the different transcriptomic and proteomic datasets.

By default, **the output page** displays the top 20 neXtProt entries but users can change this setting as they deem appropriate. The result is displayed in the form of a table (Figure 2A) containing one protein entry per line with columns for: neXtProt IDs (hyperlinked to the neXtProt knowledgebase); gene symbols (hyperlinked to the HGNC database (24)) and protein descriptions; NCBI Entrez gene IDs (hyperlinked to the NCBI website); the color-coded evolution of the neXtProt PE status over time; the current PE status; the observability status according to the classification published by Farrah *et al.* (18); the rank of each neXtProt entry computed by the PepPSy prioritization system based on the weight scheme defined by the user; and the human tissues in which the corresponding gene products display the highest abundance based on the six distinct transcriptomic and proteomic datasets (NCBI-UniGene, Affymetrix 3′ array and All Exon, Illumina RNA sequencing, HPA antibody-based and HPM LC/MS-based protein expression profiles) (Figure 2A). Results can be exported as an archive file containing both a complete (full) and a lighter (light) text files (tabular format) reporting the entire protein list and corresponding ranking information via a ‘click here’ link at the top of the page and subsequently imported into an Excel sheet.

In addition to the output table, a graphical interactive interface (Figure 2B), called Body map, is conveniently available by clicking on the stickman to the left of each neXtProt entry (Figure 2A). This tool may help users to summarize and explore the current knowledge regarding the expression at the transcript- and protein-level of each individual gene product in all major tissues of the human body through the six transcriptomic and proteomic technologies hosted in the system. Briefly, a menu on the right side contained 3 levels in which HECs (level 3, blue and italic font style) are organized in human tissues/organs (level 2) classified in major systems of the human body (level 1). Human systems, tissues and HECs are color-coded according to the expression levels of the considered gene product, measured by the six transcriptomic and proteomic technologies and displayed as first, second and third quartile values (Figure 2B) (cf. Design and Implementation). The expression values of a gene product at the system level (level 1) are inferred from the maximum expression values observed in the tissues (level 2) belonging to the same system which themselves are inferred from the maximum expression values measured in the HECs (level 3) belonging to the same tissues.

Finally, it is worth mentioning that the welcome page includes a link to a brief tutorial for PepPSy.

## Design and implementation

### Transcriptomic and proteomic data pre-processing

For each neXtProt entry in each human experimental condition (HEC), the number of ESTs from the UniGene dataset (5), intensity values from the Affymetrix 3′ Gene array and All Exon datasets (25), FPKM values from the RNA-seq dataset provided by HPA (4) and protein expression levels provided by Kim and coworkers in the HPM database (20) were extracted from the different datasets, log2-transformed and quantile-normalized to facilitate comparison between HECs. Staining intensity information provided by HPA were associated with staining intensity values of 0, 1, 2 or 3 for not-detected, low, medium or high staining intensities, respectively.

Finally, the resulting transcriptomic and proteomic expression values were discretized into four levels using quartile values: low (< first quartile, Q1), medium (< Q2), high (< Q3) and very high (> Q3).

### Weight scheme and overall prioritization

The prioritization parameters enable users defining their own weight combination or scheme reflecting the contributions of each module to the overall prioritization. This highly supple feature allows for complex queries pertaining to very specific biological questions.

As already described in the gene prioritization system (GPSy) (19), the integration of transcriptomic and proteomic datasets with distinct ranking strategies forms the basis of PepPSy’s modular architecture allowing for maximum query flexibility. In PepPSy, the precomputation of module-wise ranks greatly accelerates the process of prioritization. To combine the ranking output of each individual module, the absolute rank of each neXtProt entry in a ranked list (module *i*, protein *j*) is transformed into relative ranks using the formula:

$${rR}_{i,j=\frac{r_{i,j}}{R_{i}}}$$

where *r_i,j_* and *rR_i,j_* are the absolute and relative ranks of the protein respectively, and *R_i_* is the total number of entries in the ranked list.

When the system is queried, candidate protein entries in the input list are mapped onto the pre-computed ranked lists. An overall rank $\overset{-}{{rR}_{j}}$ of a given neXtProt identifier *j* is computed based on an inter-module weighted average of the individual module ranks:

$$\overset{-}{{rR}_{j}}=\frac{\sum_{i=1}^{I}w_{i}\times {rR}_{i,j}}{\sum_{i=1}^{I}w_{i}}$$

where *w_i_* is the weight applied to each module selected in the user interface. The final output is a reordered list based on the overall ranking of each gene product entry.

### Technical issues and updates

Above the PepPSy database generated by Python and Tcl/Tk scripts, there is a web application implemented in PHP with the Symfony framework. The application indexes the input files using the Lucene library. This index allows to search in the whole elements with multiple input cross parameters. When user selects information, the application searches in the index for matching gene product identifiers and calls the Tcl/Tk scripts with the selected prioritization parameters and the matching proteins. Results (JSON format) are then returned and displayed as a table.

As PepPSy is closely related to the neXtProt knowledgebase, information for each gene product entry is updated and processed every time a novel neXtProt release is made available to the community. Note that PepPSy offers the possibility to query the system and to prioritize lists of gene products based on the information of the older neXtProt releases.

## Application to PE5 protein reannotation

The PepPSy interface was used to search for solid transcript expression information relative to the 616 entries labelled as ‘dubious’ (PE5) in the neXtProt release 2014-09-19. In the Filtration ‘tab’, we selected 2014-09-19 as release date, PE5 as neXtProt protein evidence, and the four transcriptomics datasets. Default parameters were used for the ‘Prioritization’ step. The result was ten entries for which there was information in all the four transcriptomics datasets. Table 1 shows the prioritized list of these ten entries, with the corresponding gene symbols, observability status, and the two tissues in which they have the highest expression level according to each dataset.

**Table 1.** List of the ten PE5 entries from neXtProt release 2014-09-19 that have expression information in the four transcriptomic datasets

	neXtProt accession	Gene symbol	Observability status	UniGene	Affymetrix U133 Plus 2.0	Affymetrix All Exon 1.0	Hum. Protein Atlas RNA-seq
1	NX_B1AH88	TSPO	observable	Intestine > Brain > …	Mouth > Bone marrow > …	Spleen > Intestine > …	Bone marrow > Skin > …
2	NX_Q9NPU4	C14orf132	with special handling	Brain > Testis > …	Brain > Spinal cord > …	Brain	Brain > Oviduct > …
3	NX_P0CF97	FAM200B	likely unobservable	Brain > Eye > …	Male germ cell > Brain > …	Brain	Ovary > Kidney > …
4	NX_Q9BWV7	TTLL2	likely unobservable	Testis > …	Male germ cell > Testis	Testis	Testis
5	NX_Q5T036	FAM120AOS	likely unobservable	Brain > Lung > …	Placenta > Pituitary gland > …	Intestine > Kidney > …	Placenta > Thyroid > …
6	NX_Q96SF2	CCT8L2	likely unobservable	Testis > Brain > …	Male germ cell > Testis	Intestine > Pancreas > …	Testis
7	NX_Q8IVY1	C1orf210	with special handling	Pancreas > Intestine > …	Female germ cell	Intestine > Kidney > …	Intestine > Stomach > …
8	NX_P0CB46	CASP16	likely unobservable	Spleen > Uterus > …	Female germ cell	Intestine	Intestine
9	NX_Q8N5Q1	FAM71E2	observable	Testis > Thymus > …	Male germ cell	Testis	Testis
10	NX_Q96HZ7	C21orf119	likely unobservable	Intestine > Prostate > …	Testis > Male germ cell > …	Testis > Kidney > …	Skeletal muscle > Thyroid > …

The list has been prioritized using the default parameters of PepPSy. The four last columns show the two tissues in which the highest expression levels have been reported in each dataset.

The first entry on this list (NX_B1AH88) corresponds to a very unusual annotation case in UniProtKB/Swiss-Prot (hence neXtProt). Although the usual UniProtKB/Swiss-Prot procedure is to merge all the splice isoforms that arise from one gene into a single UniProtKB/Swiss-Prot entry, this particular isoform has been annotated as a separate entry because it results from another reading frame and does not share any sequence with the other isoform (NX_P30536) (26). Because NX_B1AH88 and NX_P30536 are potentially transcribed from the same gene, they are mapped to the same Ensembl gene identifier (ENSG00000100300) and inherit any transcriptomics data linked to this gene identifier. Therefore, the data that was retrieved for the dubious NX_B1AH88 isoform is probably an artefact and would need to be remapped to the well-known isoform (NX_P30536, PE1).

The second entry from the list, NX_Q9NPU4, corresponds to the C14orf132 gene. There is a complete consensus across the four transcriptomics datasets showing that C14orf132 is expressed at highest levels in the brain. Initially, the annotation resources had predicted that this gene would encode a 173 aa protein. After reexamination, they chose another reading frame, resulting in a 83 aa transmembrane protein, that would be conserved in most mammalian species. The sequence has been changed in UniProtKB (04-MAR-2015 release) and neXtProt (2015-04-28 release). Given the available transcriptomics data, one should look for it in brain samples, if possible after membrane enrichment. Since trypsin cleavage would lead to a single, hydrophobic peptide, a special methodology may be required for its detection. Until a conclusive proof for its existence at protein level is established, the status of the entry has been changed to PE3 (validated by homology).

For two other entries, NX_Q8N5Q1 (FAM71E2, line 9) and NX_Q9BWV7 (TTLL2, line 4), there is also a clear consensus among the four transcriptomics datasets, indicating highest expression levels in testis. Because cDNAs for FAM71E2 have been found in different tissues (thymus, testis and brain), the status of the entry has been changed to PE2 (validated at transcript level) in UniProtKB (04-MAR-2015 release). Since two unique peptides corresponding to FAM71E2 were identified by mass spectrometry in sperm (27), neXtProt reclassified the corresponding entry as PE1. The TTLL2 gene has a mouse ortholog, cDNAs have been found in testis, and the CCDS consortium has recently reclassified it as protein-coding. Therefore, NX_Q9BWV7 is currently under examination by UniProtKB/Swiss-Prot and neXtProt curators for upgrading to PE2 (validated at transcript level). The peptide GGLDAPDCLPYDSLSFTSR, which uniquely maps on the corresponding NX_Q9BWV7 entry has been identified in testis by mass spectrometry. Since a single peptide is not sufficient to upgrade a protein to PE1 (validated at protein level), targeted LC-SRM studies on testis, using other peptides will need to be performed.

For NX_Q96SF2 (CCT8L2, line 6), three datasets out of four show highest expression levels in testis. The CCT8L2 gene, only found in Human and Chimp, is thought to have arisen by duplication in the Hominoidea lineage after its divergence from the Cercopithecidae (28). Although some mass spectrometry information is available (20), it has not passed the stringent criteria quality of the HPP for validation. Until we gain a more conclusive proof for its existence at protein level, the status of the entry has been changed to PE2 (validated at transcript level). Given the available transcriptomics data, it would be wise to look for this protein in testis-related samples. However, its definitive validation by mass spectrometry may not be an easy task since it differs from its closest paralog CCT8L1P by only a few residues. This is probably why its observability status has been set as ‘likely unobservable’ by Farrah *et al.* (18).

For NX_Q8IVY1 (C1orf210, line 7), two of the datasets indicate highest expression levels in intestine. C1orf210 has a clear ortholog in mouse (2610528J11Rik, UniProtKB Q9CQM1) and has been identified by mass spectrometry in fetal liver, pancreas, prostate (29), breast (30) and ovary (31). It has been shown to be phosphorylated on Tyr-94 in human cell lines of various origins (32). Therefore, its status has been changed to PE1 (validated at protein level).

For NX_P0CF97 (FAM200B, line 3), the datasets indicate expression in various tissues, including brain. FAM200B is well conserved among mammals, and the CCDS consortium has recently classified it as protein coding. Although some mass spectrometry information is available (20), it has not passed the stringent criteria quality of the HPP. Until a more conclusive proof for its existence at protein level is available, its status has been changed to PE3 (validated by homology). However, given the high sequence similarity with FAM200A, a conclusive proof for its existence at protein level will be hard to find.

For NX_Q5T036 (FAM120AOS, line 5), two of the datasets indicate expression in placenta. It is classified as protein coding by the CCDS consortium, is conserved in several mammalian species, but has not been detected by mass spectrometry yet. Given the available transcriptomics data, it would be wise to look for this protein in placenta. Until a conclusive proof for its existence at protein level is available, this entry is currently under examination by UniProtKB/Swiss-Prot curators for upgrading to PE3 (validated by homology) or 4 (predicted).

For NX_Q96HZ7 (C21orf119, line 10), two datasets show expression in testis. However, C21orf119 is predicted to encode a long non-coding RNA. Therefore, NX_Q96HZ7 will remain PE5 until contradictory evidence is available.

For NX_P0CB46 (CASP16, line 8), two datasets show expression in intestine. However, a consensus has been reached between the different resources to classify it as a pseudogene (not protein coding). Therefore, NX_P0CB46 has been deleted from UniProtKB/Swiss-Prot and neXtProt.

In conclusion, the use of PepPSy as a biocuration companion tool has allowed to quickly prioritize 10 PE5 proteins for re-annotation by biocurators, among a total of 616 proteins. As shown in Table 2, two of them have been validated at protein level (C1orf210 and FAM71E2), and five have been or will be upgraded to PE1-3. For four of them, further unambiguous proof of their existence needs to be found by mass spectrometry or Ab-based proteomics. Table 2 indicates in which sample these proteins could be investigated.

**Table 2.** Summary of the reannotation of PE5 entries

neXtProt accession	Gene symbol	New PE	Sample suggestion for further analyses
NX_B1AH88	TSPO	PE5	
NX_Q9NPU4	C14orf132	PE3	Brain; membrane fraction
NX_P0CF97	FAM200B	PE3	Brain; will be difficult to distinguish from FAM200A
NX_Q9BWV7	TTLL2	PE1? (in progress)	Testis
NX_Q5T036	FAM120AOS	PE3? (in progress)	Placenta?
NX_Q96SF2	CCT8L2	PE2	Testis; will be difficult to distinguish from CCT8L1P
NX_Q8IVY1	C1orf210	PE1	
NX_P0CB46	CASP16	Deleted	
NX_Q8N5Q1	FAM71E2	PE1	
NX_Q96HZ7	C21orf119	PE5	
NX_Q8IYS8	BOD1L2	PE2	Testis
NX_P0CG32	ZCCHC18	PE3	Brain?; will be difficult to distinguish from ZCCHC12
NX_C9J798	RASA4B	PE3	Skeletal muscle; quasi undistinguishable from RASA4

We will continue to provide UniProtKB/Swiss-Prot curators with transcriptomic evidence and other available information for all PE5 entries with transcriptional information in at least one of the available transcriptomics datasets (433 entries in total). We have started the process with the 11 PE5 entries which have RNA-seq information in HPA and EST information in UniGene, as well as information in one of the two microarray datasets. Following this work, the status of three entries (NX_Q8IYS8, BOD1L2; NX_P0CG32, ZCCHC18 and NX_C9J798, RASA4B) has been upgraded to PE2-3. According to the three datasets, BOD1L2 highest levels are found in testis. Interestingly, BOD1L2 was unambiguously identified by two peptides detected by mass spectrometry in spermatozoa samples (27). Therefore, the entry will probably be upgraded to PE1 soon. For ZCCHC18 and RASA4B, there is no clear consensus between the transcriptomic datasets, and even with the right sample, it will be difficult to unambiguously validate their existence at protein level due to strong similarity with ZCCHC12 (NX_Q6PEW1) and RASA4 (NX_O43374), respectively.

## Conclusion

PepPSy has been developed as a user-friendly gene expression-based prioritization system, to help investigators to determine in which human tissues they should look for an unseen protein and curators to quickly look at available transcriptomics data for a list of protein. In this work, PepPSy has been applied to prioritize twenty-one proteins annotated as ‘Uncertain’ (PE5) in UniProtKB/Swiss-Prot and neXtProt for revision. As a result, 21 proteins have been provided transcriptomic evidence and biocurators have changed the status of eight of these based on all available information. PepPSy can now be used to choose the samples in which to look for the seven proteins that have been reclassified as PE2 or PE3, and to identify potentially problematic cases. In the near future, PepPSy will be used to revise the annotation of the 412 remaining PE5 entries with transcriptional information in at least one of the available transcriptomics datasets. Therefore, PepPSy has been revealed as an efficient companion tool for neXtProt biocuration and quality management workflows, and for the C-HPP project which aims to get an accurate picture of the validation status of all human protein coding genes. In the near future we will extend the scope of PepPSy to stay abreast of rapid technological advances. We will gather other relevant datasets in PepPSy to cover other biological topics by including other tissues, specific cell types from single-cell RNA-seq data, and chemical-induced/disease-associated experimental samples. Finally, we are also currently planning to develop a community tool embedded in PepPSy that will stimulate the annotation of other missing proteins by facilitating collaborative work.

## Supplementary data

Supplementary data are available at *Database* Online.

Supplementary Data
