# Supplementary material for: PepPSy: a web server to prioritize gene products in experimental and biocuration workflows
Source: Database (Oxford). 2016 May 12;2016:baw070. doi: 10.1093/database/baw070 (PMC4865363; doi:10.1093/database/baw070)
Supplement: Supplementary Data [file supp_2016_baw070_index.html]

Supplementary Data 

# PepPSy: a web server to prioritize gene products in experimental and biocuration workflows

## Supplementary Data

files

- Supplementary Data - xlsx file
